# A lifestyle intervention study targeting individuals with low socioeconomic status of different ethnic origins: important aspects for successful implementation

**DOI:** 10.1186/s12889-017-4592-1

**Published:** 2017-07-25

**Authors:** Dorit Teuscher, Andrea J. Bukman, Marleen A. van Baak, Edith J. M. Feskens, Reint Jan Renes, Agnes Meershoek

**Affiliations:** 1 NUTRIM School for Nutrition and Translational Research in Metabolism, Department of Human Biology and Movement Sciences, Maastricht University Medical Centre+, P.O Box 616, 6200 MD, Maastricht, The Netherlands; 2 Division of Human Nutrition, Wageningen University, P.O Box 17, 6700 AA, Wageningen, The Netherlands; 3 Division of Strategic Communication, Wageningen University, P.O Box 8130, 6700 EW, Wageningen, The Netherlands; 4 CAPHRI, Department of Health, Ethics and Society, Maastricht University Medical Centre+, P.O Box 616, 6200 MD, Maastricht, The Netherlands

**Keywords:** Implementation, Fidelity, Adaptation, Lifestyle intervention, Socioeconomic status, Ethnic minorities, Qualitative methods

## Abstract

**Background:**

Evaluation of the implementation process of trials is important, because the way a study is implemented modifies its outcomes. Furthermore, lessons learned during implementation can inform other researchers on factors that play a role when implementing interventions described in research. This study evaluates the implementation of the MetSLIM study, targeting individuals with low socioeconomic status of different ethnic origins. The MetSLIM study was set up to evaluate the effectiveness of a lifestyle programme on waist circumference and other cardio-metabolic risk factors. The objective of this evaluation was to identify components that were essential for the implementation of the MetSLIM study and to inform other researchers on methodological aspects when working with inadequately reached populations in health research.

**Methods:**

In this evaluation study the experiences of health professionals, study assistants, a community worker and regional research coordinators involved in the MetSLIM study were explored using semi-structured interviews. Questionnaires were used to evaluate participants’ satisfaction with the lifestyle intervention.

**Results:**

Our analyses show that a flexible recruitment protocol eventually leads to recruitment of sufficient participants; that trust in the recruiter is an important factor in the recruitment of individuals with low socioeconomic status of different ethnic origins; and that health professionals will unavoidably shape the form of intervention activities. Furthermore, our evaluation shows that daily practice and research mutually influence each other and that the results of an intervention are a product of this interaction.

**Conclusions:**

Health promotion research would benefit from a perspective that sees intervention activities not as fixed entities but rather as social interaction that can take on numerous forms. Analysing and reporting the implementation process of studies, like in this evaluation, will allow readers to get a detailed view on the appropriateness of the (intended) study design and intervention for the targeted population. Evaluation studies that shed light on the reasons for adaptations, rather than describing them as deviation from the original plan, would point out methodological aspects important for a study’s replication. Furthermore, they would show how various factors can influence the implementation, and therewith initiate a learning cycle for the development of future intervention studies.

**Trial registration:**

Netherlands Trial Register NTR3721 (since November 27, 2012).

## Background

In recent years, much emphasis has been placed on studying the effectiveness of lifestyle interventions – already shown to be effective in a clinical or an academic setting – in routine primary care and among diverse target groups [1]. In the Netherlands, the SLIM study, a lifestyle intervention promoting a healthy diet and increased physical activity, decreased diabetes risk and risk of metabolic syndrome in a university setting [2, 3]. In the SLIM study, individuals with low socioeconomic status (SES) dropped out more than individuals with higher education [4]. High dropout rates have also been reported in other lifestyle intervention studies [5] and are also observed in studies targeting ethnic minorities [6]. Additionally, these groups are less likely to participate in lifestyle interventions in the first place [7, 8]. This is worrisome, as a higher prevalence of cardio-metabolic diseases is observed in these inadequately reached groups [9, 10], and to date in the Netherlands there is no effective lifestyle intervention targeting cardio-metabolic risk in low SES adults [11].

Therefore, the MetSLIM study, which targets low SES individuals of different ethnic origins, was set up to evaluate a version of the SLIM lifestyle intervention adapted to the needs and preferences of this group [12]. The adaptations were based on preceding research focusing on the identification of group members’ needs and preferences [13, 14]. The research team made certain choices on the basis of the gathered information, such as adding extra group meetings about topics relevant for the target group; involving ethnicity- and gender-matched research assistants, dieticians and sports instructors; providing activities for women and men separately; and setting up all activities in participants’ own neighbourhood. Changes to the study design, setting and measurements were required because of the adaptations in the lifestyle intervention programme and in order to minimise the burden of participation [12]. Between January 2013 and June 2015, this quasi-experimental study was implemented in two cities in the Netherlands. The MetSLIM lifestyle intervention was effective in improving obesity-related measures such as waist circumference, waist-to-height-ratio (WHtR), body weight, fat percentage and BMI after one year (Bukman AJ, Teuscher D, Meershoek A, Renes RJ, van Baak MA, Feskens EJM. Effectiveness of the MetSLIM lifestyle intervention targeting low SES individuals of different ethnic origins with elevated waist-to-height ratio. submitted. 2016.).

Great emphasis is often placed on these biomedical outcomes of trials like the MetSLIM study and their reproducibility. For this reason, researchers aim to standardise intervention components that are, in the best case, delivered uniformly to the target population. However, it has also been argued that, if health professionals are involved in such lifestyle intervention studies, they may tailor intervention components according to their own ideas and that prevention work is hard to standardise and to control [15]. Researchers have pointed out that generally a tension exists between fidelity and adaptations to the target group’s needs, when translating evidence-based interventions into practice [16].

Common process evaluations however identify the conditions under which the intervention is deemed to be effective and the deviations from the original study protocol [17], not seldom concluding that a lack of effectiveness was the result of low attendance rates of participants and/or insufficient commitment or skills of health care professionals [18, 19]. This perspective portrays a common view in health research that a designed intervention should not be altered and eventually non effectiveness is a result of inadequate implementation of the designed intervention, rather than questioning if the methods used were adequate to achieve one’s goal. Researchers have argued that evaluators have to move beyond this simplistic documentation of implementation fidelity in order to understand how a programme worked or not [20, 21]. Considering changes to the original study design and intervention as integral part of implementation, rather than as confounder will enable us to understand what happens in the practice of implementing health promotion research. This view will also offer researchers and practitioners with a guide of factors that play a role when implementing intervention programmes.

Therefore, the aim of this evaluation study is to reveal important aspects in the implementation process of the MetSLIM study that will show if the study design and intervention were appropriate for the targeted population, which challenges were faced during implementation and which changes were made for which reasons. The outcome of this evaluation could guide the development of future studies and advise on appropriate methodological approaches with respect to (lifestyle) intervention research targeting a generally inadequately reached population in health research.

## Methods

### The initial MetSLIM study design

The MetSLIM study was set up in order to evaluate the effectiveness of the adapted SLIM lifestyle intervention targeting low SES individuals of different ethnic origins. Here, we describe the design of the study as originally planned. A more detailed description can be found in Teuscher et al. [12]. The aim was to recruit people with a WHtR above 0.5, who were between 30 and 70 years old, were not taking medication for hypertension, hypercholesterolemia, cardiovascular diseases, diabetes mellitus and/or renal failure at baseline, and were of Dutch, Turkish or Moroccan ethnic origin [12]. Participants’ ethnicity was categorised as Dutch if both parents were born in the Netherlands [22] and as Turkish/Moroccan if at least one parent was born in Turkey/Morocco [23]. People who had a mental or physical disability that made participation in a lifestyle intervention impossible, were already participating in a lifestyle programme targeting weight loss, or were pregnant or lactating were excluded.

Participants were recruited either through their general practitioner (GP) or at community centres located in socioeconomically deprived neighbourhoods, as these were the target group’s preferred channels as identified in previous research [24]. Therefore, the researchers recruited GPs situated in socioeconomically deprived neighbourhoods or with a broad spectrum of low SES and/or ethnic minority patients. These GPs selected potential participants on the basis of the inclusion criteria in their database, e.g. postal code (as indicator for neighbourhood), age and medication use. Furthermore, the GPs were asked to select only patients that they knew to be of Dutch, Turkish and Moroccan ethnic origin and who were physically and mentally able to participate in the intervention. Because waist circumference was not in the GPs’ database, this could not be used as a selection criterion. Thus, selected potential participants received an invitation letter containing a brief screening questionnaire/registration form, an information booklet about the study, a tape measure and a return envelope. They were asked to return the screening questionnaire/registration form with information about self-measured waist circumference and height in the return envelope to the researchers if their WHtR was above 0.5 and they were interested in participating. Furthermore, the researchers contacted community health workers (e.g. social workers), local health professionals and other local contacts in order to promote the intervention study in community centres.

The same measurements (anthropometrics, blood and urine analysis, questionnaires on physical activity, food consumption and quality of life, accelerometry) were performed in both the intervention and the control group at baseline and after 12 months. The intervention group received a 12-month lifestyle programme promoting lifestyle change and moderate weight loss. The lifestyle intervention aimed to facilitate an increase in physical activity and a change in dietary habits in line with the national guidelines on healthy nutrition [25]. Therefore, the three main components of the MetSLIM lifestyle intervention were: four group meetings and four hours of individual dietary advice and weekly one-hour physical activity lessons. The group meetings were guided by an ethnicity-matched dietician and focused on familiarisation with one another, label reading, social occasions and price concerns (supermarket tour). These dieticians, who also provided a maximum of four hours of individual dietary advice to participants, were asked to divide the four hours over a flexible number of consultations and to tailor their advice, based on the national guidelines on healthy nutrition, to the participants’ needs. All activities were provided in a community setting in the participants’ neighbourhood in order to facilitate social cohesion among participants. The research coordinators developed a manual for the research assistants, the dieticians and the sports instructors, so that core elements of the MetSLIM intervention would be delivered to the target population despite the different locations and professionals involved.

The control group participants attended one group meeting at the start of the study in which a dietician (if necessary, accompanied by a language assistant with a dietetic background) provided general information about a healthy diet. Furthermore, control group participants received information leaflets on the beneficial effects of a healthy diet and sufficient physical activity.

The MetSLIM study was approved by the Medical Ethical Committee of Wageningen University and registered at the Netherlands Trial Register as NTR3721. All participants gave their written informed consent before the start of the study.

### Data collection

Data for this evaluation study were gathered from health professionals, who delivered the intervention to the participants; study assistants, who helped with recruitment and measurements; a community worker, who was involved in the recruitment; and two regional research coordinators, who coordinated the MetSLIM intervention in the local setting. Semi-structured interviews were held with all dieticians (six) and all sports instructors (six) involved in the MetSLIM study, with three of the five study assistants, and with one community worker, all with their audiotaped consent. The two regional research coordinators (first two authors of this article) were interviewed by a master’s student.

Furthermore, data of participants in the MetSLIM study were used for this evaluation study. At the end of the intervention study, participants were given a questionnaire regarding satisfaction with the lifestyle programme, with the consultations with the dietician, with the group meetings and with the physical activity lessons on a scale of 1–10 (10 being the best score). Furthermore, the regional research coordinators’ and the health professionals’ attendance registration records were used to assess compliance with intervention components (dietary consultations and group meetings).

### Data analyses

For this evaluation study, descriptive statistics of the quantitative data were obtained using SPSS Statistics (version 23). Unfortunately, the registration records of the physical activity lessons were not complete and could therefore not be used for further analysis.

The interviews with the health professionals, the regional research coordinators, the research assistants and the community worker were tape-recorded and transcribed verbatim. Transcripts were analysed using an inductive approach [26]. In each transcript descriptive codes were connected to frequently emerging themes. These codes were then grouped into broader categories. These broader categories were: recruitment, group meetings, individual dietary advice and physical activity lessons. These categories were used as the coding scheme to analyse the qualitative data and interpret on what worked well and which problems were encountered during the implementation of the MetSLIM study. In this sense, this evaluation provides detailed answers to the following questions:

1. What direction did the intended recruitment plan take and what lessons were learned?
2. How were the group meetings with relevant topics for the target group organised and experienced by the dieticians and the target group?
3. How did the dieticians shape individual dietary advice during the course of the intervention and what were their experiences?
4. How were the MetSLIM intervention’s physical activity lessons concretised in the community setting and experienced by sports instructors?

## Results

In this section, we describe the findings in relation to the implementation of the MetSLIM study: (1) recruitment period, (2) group meetings, (3) individual dietary advice and (4) physical activity lessons.

### Evaluating the recruitment for the MetSLIM study

Between January 2013 and June 2014, 220 participants with elevated WHtR enrolled in the MetSLIM study. Of these, 117 were recruited for the intervention group and 103 for the control group. More than half of them (54%) reported hearing of the study from their GP, and 46% reported hearing of it in community centres.

Many Turkish and Moroccan women were recruited by word of mouth. Once women heard of the (free) physical activity lessons in the community centre, they went to the lessons and there they were asked if they were willing to participate in the MetSLIM study if they were eligible. The majority of the study participants were female (81%), 36% were of Dutch ethnic origin, 49% of Turkish ethnic origin, 7% of Moroccan ethnic origin and the remaining 8% of different ethnic origins. The mean age of the recruited study population was 46.6 (SD 9.6) years. Forty per cent of participants had not attended school, or only primary school. A detailed description of the study participants can be found elsewhere.

The MetSLIM study used two recruitment strategies that had been identified by the target group as preferred ways in preceding research [14, 24]: to recruit participants, firstly, via GPs situated in socioeconomically deprived neighbourhoods and, secondly, via community centres also situated in socioeconomically deprived neighbourhoods. However, the regional research coordinators encountered difficulties in finding GPs willing to help with the recruitment of the target group.

*I think they [GPs] are just really busy, that’s the problem, and they don’t have time – I mean, patients say they [GPs] don’t have time for the patients, but [GPs] don’t like it themselves, that they don’t have the time [for their patients] (…) then, research is just not their first priority. (Research coordinator 1)*

Eventually, 11 GPs were recruited who were willing to invite patients to participate in the MetSLIM study. Researchers reported additional challenges in the recruitment of low SES individuals of different ethnic origins through GPs. First of all, using the selection criteria to select potential participants from the GP records was very time-consuming. Secondly, some GPs did not select patients strictly on the inclusion and exclusion criteria provided by the researchers, but rather according to their personal opinion of who needed the intervention most.

*My experience was that one of our GPs said to herself ‘okay I think that person needs it and that person is overweight’ and I thought ‘no please use our selection criteria’ because I think it just ended up that she mainly selected those [patients] that were overweight and she thought they would need this kind of intervention. (Research coordinator 2)*

Selecting patients through the GPs’ database was additionally hampered, as ethnicity is not registered by GPs in the Netherlands. This resulted in patients of ethnic origins other than Dutch, Turkish or Moroccan being invited. However, excluding patients after they had been invited by their GP resulted in dissatisfaction among those patients, as one of the research coordinators recounts:

*They were already invited by their GP and selected by their GP so who are we [as researchers] to say ‘okay, they [the GP] invited you, but we do not want you to join’ (…) that is actually the message you give them. (Research coordinator 2)*

The same challenge was faced recruiting people in community centres. Because of issues surrounding discrimination, the exclusion of ‘other’ ethnicities was sensitive and was eventually dropped by the research team. A similar situation arose for inclusion based on postal code. It did not make sense to recruit people in community centres and exclude people with a (nearby) postal code from an intervention that aimed at social cohesion. Additionally, the research coordinators and assistants were dependent on third parties for the recruitment via community centres, as this research assistant reports:

*Ehm, so then we often looked at the community centres of interest, and you were so often dependent on the local professionals, also the social workers that were willing to help you to connect with certain groups. (Research assistant 1)*

In the recruitment process, trust proved to be a very important factor specifically for ethnic minorities, as this research assistant explains:

*That there is a trusted person who they know, that there is someone involved in the study who they trust. That is the most important. I think that is the most important when recruiting people. (Research assistant 2)*

The use of ethnically matched research assistants helped to create trust; this Dutch research assistant describes the immediate connection that the research assistant of Moroccan ethnic origin had with a group of Moroccan women that they approached:

*Because she clicked immediately, you know? With the women and they are also, it is something different compared to our culture, we are not used to hugging when we see someone for the first time, but they often did that, they kissed and yeah it’s a nice thing to see. (Dutch research assistant about Moroccan research assistant)*

Furthermore, being able to talk in their mother tongue seemed to lessen barriers among the target group, as this community worker explains:

*That really did have a positive effect. That people could just talk in their native language, that made them feel at home, they could talk about everything. (Community worker)*

Initially, the intended recruitment period for the MetSLIM study was six months. However, due to the difficulties with recruiting GPs willing to invite the target group and the time that had to be invested in recruiting via the community centres, the recruitment period had to be extended and eventually lasted 17 months. As described previously, we chose to adapt the inclusion and exclusion criteria regarding ethnicity and postal code during the recruitment period. The aim remained to recruit people of Dutch, Turkish and Moroccan origin living in deprived neighbourhoods; however, people of other ethnic origins or people living close to the socioeconomically deprived neighbourhood were not excluded.

### Evaluating the implementation of the lifestyle intervention

#### Group meetings on nutrition advice

The dieticians appreciated the manual developed for the group meetings as a convenient tool to check whether they had mentioned everything during these meetings:

*Yes I like it, because everything is written down. Just a look and you know if you have said everything and you can check that you did not forget to mention some things. (Dietician 1)*

The dieticians reported that they appreciated the contact with the regional research coordinator where they could discuss problems and decisions could be taken to adapt the lifestyle intervention, like adding an additional topic for a group meeting. For instance, Dutch participants were offered the choice between an originally planned supermarket tour and a group meeting on motivation. This alternative was offered because the Dutch dieticians were concerned that low SES participants would not be willing to discuss dietary habits in the supermarket in public. For the Turkish participants, the dietician of Turkish origin chose to offer group meetings about Ramadan instead of a supermarket tour, as participants had many questions about dietary change with regard to Ramadan.

Overall, the social cohesion stimulated by the group meetings worked well according to the dieticians, as participants motivated one another to overcome barriers and achieve their goals during those meetings.

*If participants had consultations only on an individual level, they would not see what the other participants do. And in such a group [meeting] it is discussed what the others do. Extra motivation. (Dietician 2)*

*Yes, I think especially the group meetings, and the physical activity lessons together, that stimulates a lot of people, thus the more they are together, the more they are engaged [with the intervention], and that motivates them to keep going. (Dietician 3)*

In line with this, the dieticians mentioned that the frequency of the group meetings spread over the year could be higher:

*A gap of three months between group meetings is too long, participants also mention that. They say, ‘We like to see one another to exchange our experiences’. (Dietician 1)*

Of the 80 participants that completed the lifestyle intervention, 76% attended the introductory meeting and 34% attended all the following (three) group meetings. Overall, the participants gave the group meetings 7.5 (SD = 1.6) points out of 10. The mean values of the satisfaction scores are presented in Table 1.

**Table 1:** Evaluation by participants of the components of the MetSLIM lifestyle intervention ^a^

	Satisfaction Score (1–10)
Rate MetSLIM lifestyle intervention programme (*n* = 60)	8.3 (1.3)
Rate dietary consultations (*n* = 57)	8.5 (1.5)
Rate introductory meeting (*n* = 56)	7.6 (1.4)
Rate group meeting on label reading (*n* = 46)	7.7 (1.6)
Rate group meeting on social occasions (*n* = 39)	7.6 (1.7)
Rate group meeting including the supermarket tour (*n* = 14)	8.4 (1.6)
Rate group meeting on Ramadan (*n* = 11)	8.0 (1.9)
Rate group meeting on motivation (*n* = 18)	8.1 (1.2)
Rate group meetings (*n* = 54)	7.5 (1.6)
Rate physical activity lessons (*n* = 55)	8.4 (1.5)
Nice to work on lifestyle in a group (*n* = 63)	
(very) nice	82.6%
neutral	15.9%
not nice (at all)	1.6%
Nice to work on lifestyle close to my home (*n* = 62)	
I do not live close by	8.1%
(very) nice	88.7%
neutral	3.2%
not nice (at all)	0
Nice to work on lifestyle with people from the neighbourhood (*n* = 61)	
I do not live close by	6.6%
(very) nice	80.3%
neutral	13.1%
not nice (at all)	0
Nice to make new contacts (*n* = 63)	55.6%

^a^Values are expressed as mean ± SD or %

#### Individual dietary advice

On average, participants had 123.5 min (SD = 71.0) of individual consultations with the dietician spread over 12 months; this was less than the 160 min that should have been offered according to the protocol. Overall, the participants reported being satisfied with the individual dietary advice. Participants (*n* = 56) gave the consultations 8.5 (SD = 1.5) points out of 10.

The dieticians reported following the manual with regard to the individual consultations to a lesser extent. Instead, they often applied their own professional skills. They appreciated the flexibility to tailor the content of the individual consultations to each participant’s needs. In the manual, it was requested to use Motivational Interviewing. The dieticians described the use of Motivational Interviewing more as a general attitude, rather than an approach that they did or did not apply. They mentioned that they tailored the advice and the manner in which they gave that advice to each participant’s needs and skills:

*[On Motivational Interviewing] Yes, everyone has different communication skills, everybody is different and with some participants you make more use of it than with others. (Dietician 1)*

The dieticians noticed that the target group had a high threshold regarding self-mobilisation to take action. Dietician 3 explains that generally they only showed initiative when someone, like their GP, told them to do something, in this case make an appointment with the dietician:

*Yes, they say they are going to do it [getting a referral letter for the dietician], but they really just get into action when the GP says so. […] Often you see that they do not take the initiative. But when the GP says ‘you have to lose weight, you have to go to the dietician’, then they get into action. (Dietician 3)*

The dieticians’ constant monitoring of the individual consultations seemed to help to motivate the participants. However, this requires a lot of effort. This dietician explains how she perceived that she had to animate participants:

*Yes, that they forget the [individual dietary] appointments. I got the feeling that the healthcare professionals had to push them. That is important. But I had expected that if you offer them this [lifestyle intervention], and you provide group meetings and you teach them, that they themselves get into action. They got into action, but after all with a little bit of pushing, like ‘come on’. They seem to need that. (Dietician 4)*

### Physical activity lessons

For the weekly physical activity lessons, venues in the community were hired by the research coordinators in consultation with the sports instructors. The sports instructors reported that they found the instruction manual helpful at the beginning of the study, as it helped them to know what was expected of them. Furthermore, they especially appreciated that they could always contact the regional research coordinators to adjust time schedules and discuss other issues that arose during the physical activity lessons.

These lessons were provided separately for women and men with gender-matched sports instructors, because preceding research had indicated that the target group preferred this [14]. However, the Dutch participants in the MetSLIM study indicated when enrolling in the sports lessons that they did not mind participating in a mixed gender group. Therefore, the physical activity lessons for Dutch participants were provided for women and men together. The satisfaction score for the physical activity lessons was 8.4 (SD = 1.5) out of 10 points. The tailoring of the physical activity lessons was an on-going process; once the sports instructors knew the group’s preferences, they adapted the lessons accordingly:

*Sometimes they say ‘I like this’ and then I do it again in another lesson. (Sports instructor 1)*

Furthermore, participants were allowed to bring friends and family members along to the physical activity lessons for motivational purposes. However, this was not possible at one of the three physical activity locations as the room was too small to offer this. The sports instructors report that especially the emphasis on social cohesion made it a lot easier for participants to go to the physical activity lessons:

*It is always difficult to go [to the physical activity lessons], I mean when you work the whole day or you have been busy with something else and the weather is nice, then it is always difficult to go. But they feel the group cohesion, that they are close with one another. That is why they come to the lessons, because they find it very sociable. (Sports instructor 1)*

However, the sports instructors were worried about the continuation of physical activity after the end of the one-year intervention period, as they did not see the target group taking the initiative to find a sports class on their own. Sports instructor 2 thinks the group will stop being physically active if the opportunity to continue is not provided in the same context as in the lifestyle intervention:

*Then [when the programme stops] they will also stop being physically active, I think. […] It will become more difficult, where should they go, it will cost money. They might do it on their own for a month but then it stops. Unless they could go somewhere together as a group so that they can continue the same way if this programme is no longer offered. (Sports instructor 2)*

## Discussion

The quantitative findings of the Met SLIM study demonstrate that the lifestyle intervention can be effective in improving obesity-related measures such as waist circumference, waist-to-height-ratio (WHtR), body weight, fat percentage and BMI after one year. The aim of this evaluation study was to investigate essential aspects that had potential impact on the successful implementation of the MetSLIM study. The study and lifestyle intervention was thoughtfully designed to meet the needs of the target group. However, the findings reported in this evaluation study reveal three main aspects with regard to methodological approaches that had a fundamental impact on the successful reach of the target group and the implantation of this study in deprived neighbourhoods.

Firstly, our results show that rigid inclusion and exclusion criteria can hinder the recruitment of underserved populations in health research. The inclusion and exclusion criteria in the MetSLIM study were eventually made more flexible in order to recruit the target group and to avoid unease among people involved in recruitment. One can generally question whether the ambition to recruit a homogenous target group that complies with strict inclusion and exclusion criteria in order to prove effectiveness in a certain population is not at odds with the needs of the usual care setting treating a broad variety of patients. Researchers have argued that strict eligibility criteria can limit the external validity and generalisability of trials [27, 28]. In line with this, other researchers argue that it is not of much value for the real world to find treatment effects in a small and narrow subgroup, defined by multiple exclusion criteria [29, 30]. Additionally, as one of the core elements of this lifestyle intervention was sociability and group cohesion, the inclusion and exclusion criteria disturbed the recruitment process and might even have had a negative influence on retention if participants’ friends and family members had been excluded from the study. Furthermore, recruitment criteria based on ethnicity are ethically sensitive. The definition of ethnicity is ambiguous, and ‘ethnicized’ research practices reinforce the perception of biological race and the relationship between biological race and health behaviour [31]. However, in order to be able to tailor intervention components to local languages, this has to serve as a starting point. Similar to our case, e.g. adapting inclusion and exclusion criteria regarding ethnicity and postal code, in another study the dropping of the inclusion criteria on medication treatment eventually resulted in recruitment of sufficient participants [32]. Researchers should be aware that they are unlikely to be able to fully anticipate the endless circumstances that arise during the actual recruitment process.

Secondly, this evaluation indicates that trust in the recruiter is an important factor in the recruitment of low SES individuals of different ethnic origins and should be considered an important methodological factor when inadequately reached populations are being recruited. Like in another study, our ethnicity-matched recruiters immediately generated a feeling of trust and familiarity among potential participants [33]. Other researchers have reported the ‘attractiveness’ of a recruiter as an important factor for successful recruitment [32]. In Chang et al.’s study, participants also mentioned that positive comments by their GP or trusted clinician motivated them to participate in the study. In line with this, the recruitment channels for the MetSLIM study seemed appropriate strategies, as the GP is a trusted person and the community centre is already a familiar setting for potential participants. Nevertheless, the recruitment was extremely time-consuming, also because of the dependence on third parties with other priorities (GPs, community workers).

Thirdly, our results show that GPs and health professionals have their own agenda, and researchers might have different goals and opinions compared to health professionals. Healthcare professionals are thus co-producers of intervention components and their outcome [34]. This can endanger the execution of a trial in the way it has been designed, and a tension arises between scientific rigour and contextual fit. In research trials, healthcare professionals are often reduced to instruments necessary for conducting research, and sometimes they are even blamed for the ineffectiveness of health promotion programmes [15, 35]. Our results emphasise that, in complex lifestyle interventions, a standardised approach that values health professionals’ experiences and opinions and allows form adaptations will result in an intervention that is responsive to the needs of the target population. The crucial difference lies in the way standardisation is defined [36]. In order to evaluate complex interventions at multiple sites, the fixed aspects of an intervention should be the essential functions (core elements), like the delivery of nutrition education tailored to the local context and the individual, rather than delivering exactly the same information to every participant. Thus, the aim should be to standardise the core components (functions) of the intervention, but to allow the manner (form) to be adapted to the prevailing needs and circumstances. In line with this, it has been pointed out that professional care is a matter of tinkering and adjusting care to patients’ circumstances [37]. Care requires ‘a sensitivity to sensible compromising’ [37] and cannot – or even should not – be standardised. An approach that gives this autonomy and responsibility to health professionals will result in a trial that does not hinder real effects from happening and therefore creates an intervention that will be more easily translated into daily practice. However, researchers should keep a continuous watch on adaptations made by health professionals, because significant changes in the programme’s core components might endanger the objectives of the trial and no longer lead to the desired outcomes [38]. In our case, the regional research coordinators ensured that the core elements of the MetSLIM intervention were implemented and noticed that too little interference can actually endanger the data collection and process of a trial.

The methods for this evaluation study purposefully places interviews at the centre of the implementation analysis, rather than focusing on quantitative data. A possible limitation of the qualitative data is that the interviewers were well known by the interviewees. We however experienced that this led to very open communications, rather than to social desirable answers.

To conclude, this evaluation study shows that intervention development and implementation are intertwined. Because of the dynamics of daily practices, the development process of interventions when embedded in different settings will never be over [34]. A continuous dialogue between researchers and health professionals will therefore establish reciprocal relations and enable researchers to pay attention to the social dynamics and shifting circumstances when implementing an intervention [39]. This requires flexibility on the part of research teams and health professionals in order to adapt quickly to changing local circumstances and underlines the importance of continuous evaluation and practical reflection. Tightly controlled health research might produce significant findings. However, these findings will be of less practical value for daily practice, as the conditions that have been created for such trials cannot be reproduced in settings in which the intervention would actually be carried out in real life.

## Conclusion

Our study shows that a lifestyle intervention study can be implemented under conditions that are not strictly predefined and highly controlled and that local context shapes the implementation of lifestyle interventions. Furthermore, our results demonstrate that researchers should think carefully about their study design, as methodological aspects likewise influence the implementation of an intervention. In the end, the results of a trial are a reflection of the intertwined aspects of the intervention, the research and the local context.

## Abbreviations

GP: general practitioner
SES: socioeconomic status
WHtR: waist-to-height ratio
